# Metabolic risk factors of ovarian cancer: a review

**DOI:** 10.5935/1518-0557.20210067

**Published:** 2022

**Authors:** Neda Khanlarkhani, Elham Azizi, Fardin Amidi, Mahshad Khodarahmian, Ensieh Salehi, Azar Pazhohan, Bagher Farhood, Keywan Mortezae, Nasser Hashemi Goradel, Maryam Shabani Nashtaei

**Affiliations:** 1 Department of Physiology and Pharmacology, Karolinska Institute, Sweden; 2 Department of Biology and Anatomical Sciences, School of Medicine, Shahid Beheshti University of Medical Sciences, Tehran, Iran; 3 Department of Anatomy, School of Medicine, Tehran University of Medical Sciences, Tehran, Iran; 4 Infertility department, Arash Women's Hospital, Tehran University of Medical Sciences, Tehran, Iran; 5 Department of Gynecology, School of Medicine, Fertility and Infertility Research Center, Dr. Ali Shariati Hospital, Shahid Mohammadi Hospital, Hormozgan University of Medical Sciences, Hormozgan, Iran; 6 Infertility Center, Academic Center for Education, Culture and Research, East Azarbaijan, Tabriz, Iran. / Department of Midwifery, Urmia Branch, Islamic Azad University, Urmia, Iran; 7 Departments of Medical Physics and Radiology, Faculty of Paramedical Sciences, Kashan University of Medical Sciences, Kashan, Iran; 8 Department of Anatomy, School of Medicine, Kurdistan University of Medical Sciences, Sanandaj, Iran; 9 Department of Medical Biotechnology, School of Advanced Technologies in Medicine, Tehran University of Medical Sciences, Tehran, Iran; 10 Department of Anatomy, School of Medicine, Tehran University of Medical Sciences, Tehran, Iran. / Infertility Department, Shariati Hospital, Tehran University of Medical Sciences, Tehran, Iran

**Keywords:** ovarian cancer, diabetes mellitus, obesity, insulin resistance, inflammation

## Abstract

Ovarian cancer continues to be the leading cause of death from gynecological cancers. Despite inconsistent results, patients with metabolic abnormalities, including obesity and diabetes mellitus (DM), have poorer outcomes, showing a correlation with ovarian cancer incidence and ovarian cancer survival. Since ovarian cancer is the most common cancer in women, and considering the increasing prevalence of obesity and DM, this paper reviews the literature regarding the relationship between the aforementioned metabolic derangements and ovarian cancer, with a focus on ovarian cancer incidence, mortality, and likely mechanisms behind them. Several systematic reviews and meta-analyses have shown that obesity is associated with a higher incidence and poorer survival in ovarian cancer. Although more studies are required to investigate the etiological relation of DM and ovarian cancer, sufficient biological evidence indicates poorer outcomes and shorter survival in DM women with ovarian cancer. A variety of pathologic factors may contribute to ovarian cancer risk, development, and survival, including altered adipokine expression, increased levels of circulating growth factors, altered levels of sex hormones, insulin resistance, hyperinsulinemia, and chronic inﬂammation. Thus, obesity and DM, as changeable risk factors, can be targeted for intervention to prevent ovarian cancer and improve its outcomes.

## INTRODUCTION

Ovarian cancer is the seventh most common neoplasia and the fifth leading cause of cancer-related death in women worldwide (Ferlay *et al*., 2015). Incidence and mortality rates of the disease have been reported to be higher in more developed countries (Torre *et al*., 2015). Lower survival in ovarian cancer can chiefly be attributed to the fact that the vast majority of women with ovarian cancer is diagnosed in the advanced stage of the disease with intraperitoneal metastasis (Siegel *et al*., 2018; Protani *et al*., 2012).

Various ovarian cancer risk factors have been identified, including age, reproductive history, modifiable lifestyle factors, family history, and genetic mutations (Collaborative Group on Epidemiological Studies of Ovarian Cancer, 2008; Barry *et al*., 2014; Collaborative Group on Epidemiological Studies of Ovarian Cancer, 2012; Pennington & Swisher, 2012). Ovarian cancer cells use adipocytes as an energy source for growth and migration (Nieman *et al*., 2011). However, the prevalence of metabolic derangements, since modifiable lifestyle factors, such as obesity, type II diabetes mellitus (DM), and metabolic syndrome has grown dramatically in most parts of the world. Recent reports show that there is an association between each of these conditions and ovarian cancer (Siegel *et al*., 2018; Protani *et al*., 2012; Olsen *et al*., 2007; Shah *et al*., 2014; Lauby-Secretan *et al*., 2016).

Overweight [BMI (body mass index) ≥ 25] and obesity (BMI ≥ 30) have become epidemic worldwide, and their prevalence in women has more than doubled within the past four decades (NCD-RisC, 2016). Moreover, recent studies have also described an association between increased BMI and enhanced risk of ovarian cancer (Collaborative Group on Epidemiological Studies of Ovarian Cancer, 2008; Olsen *et al*., 2013), and reported the adverse effects of obesity on survival of women with ovarian cancer (Protani *et al*., 2012; Bae *et al*., 2014; Yang *et al*., 2011). The obesity-related comorbidities, such as type 2 DM, have also become more prevalent globally (Danaei *et al*., 2011; Kim, 2011). About 60% of adults with DM also have obesity, and 80% of them have a BMI > 25 as well (Iglay *et al*., 2016). Previous studies have indicated the relationship between DM and poorer cancer outcomes and shorter survival in women with ovarian cancer; however, the detailed link between DM and ovarian cancer is still unknown (Shah *et al*., 2014; Bakhru *et al*., 2011). Additionally, the results of a meta-analysis in 2012 showed a moderately increased risk of ovarian cancer in women with DM (Lee *et al*., 2013).

Since ovarian cancer is an associated public health issue, gaining a profound understanding of its major risk factors, especially preventable ones, such as metabolic risk factors, can be critical and should consequently be considered to develop preventive measures. Hence, the purpose of this review is to present an overview of the recent evidence regarding the association between metabolic dysregulations (obesity and type II DM) and ovarian cancer incidence and survival, and also the potential underlying biological mechanisms.

## OBESITY AND OVARIAN CANCER INCIDENCE

Cancer cells often manifest altered metabolic pathways and provide energy from fat metabolites such as fatty acids, glucose, and cholesterol that contribute to the growth of ovarian cancer. Also, fatty acids are crucial for the cell membrane and protein modification in cancer cells (Munir *et al.*, 2019). So, the excess source of energy in the human body may enhance the risk of carcinogenesis (Bianchini *et al*., 2002). It has been demonstrated that excess body weight is a preventable risk factor for several types of cancers (Calle *et al*., 2003; Renehan *et al*., 2008; Boeing, 2013). Evidence suggests the associations of ovarian cancer risk with sphingolipids, total cholesterol, triacylglycerol, and negative association of this cancer with high-density lipoproteins (Zeleznik *et al*., 2020). The association of obesity with ovarian cancer risk has been extensively evaluated by various studies. However, there is a critical need to address the inconsistent findings in the literatures (Craig *et al*., 2016).

The American Institute for Cancer Research and the World Cancer Research Fund recently declared that greater body fatness (marked by BMI) is a probable risk factor for ovarian cancer. According to the Ovarian Cancer 2014 Report (Continuous Update Project), there was an increased ovarian cancer risk of 6% per 5 BMI units, despite the substantial heterogeneity between investigated studies such as tumor type, use of hormone therapy, and menopause (Mayne *et al*., 2016). Besides, a growing body of studies has shown that increased BMI can enhance the risk of ovarian cancer (Poorolajal *et al*., 2014; Calle *et al*., 2003; Wolk *et al*., 2001; Dal Maso *et al*., 2002; Engeland *et al*., 2003; Rodriguez *et al*., 2002; Fairfield *et al.*, 2002; Nagle *et al*., 2015). This association is even highlighted by a recently published meta-analysis, indicating that the highly signiﬁcant increased risk of ovarian cancer accompanies the increased body weight in Caucasian and Asian premenopausal women. Of note, in particular, severe obesity revealed a stronger risk effect (Liu *et al*., 2015). In terms of offering a more accurate estimate of true visceral adiposity and, consequently, the risk of obesity-related cancers, the waist-to-hip ratio can be assessed instead of BMI. However, many more studies are needed for a more comprehensive analysis of this association (Renehan *et al*., 2008).

Ovarian cancer has several histologic subtypes' reﬂecting different developmental pathways (Kurman & Shih, 2008). Herein, it is worth mentioning an association that is slightly more positive for invasive endometrioid, borderline serous, and the invasive mucinous tumors with the exception of invasive serous cancer, which is the most fatal subtype (Olsen *et al*., 2013). Likewise, higher risks were reported for borderline serous tumors and invasive mucinous tumors in a meta-analysis of 47 studies (Collaborative Group on Epidemiological Studies of Ovarian Cancer, 2012). In contrast, in a population-based case-control study, the overall association was just found for clear cell subtype (Olsen *et al*., 2008). A Mendelian randomization study, evaluated a number of 39 studies of the International Ovarian Cancer Association Consortium, used genetic markers as proxies for risk factors and provided clear evidence that genetically predicted increasing BMI (per 5 kg/m^2^) was associated with enhanced risk of low grade serous ovarian cancers (Dixon *et al*., 2016). Moreover, the researchers of the latter study also found that consistent with previous observational studies, the association was strongest for low grade/borderline serous cancers (Dixon *et al*., 2016). Recently, Fortner *et al*. (2019) studied 1.3 million women with ovarian cancer and reported that the high BMI was only associated with a higher risk of highly aggressive ovarian cancer, regardless of the tumor subtype.

Remarkably, the time at which obesity progresses throughout a woman's life may be an essential risk factor for ovarian cancer. A positive association between elevated BMI in adolescence/early adulthood and increased risk for epithelial ovarian cancer has been determined by numerous studies (Dixon *et al*., 2016; Anderson *et al*., 2004; Engeland *et al*., 2003; Lubin *et al*., 2003). In addition, in a Norwegian cohort of approximately 1.1 million women, followed for an average of 25 years, an increased BMI in adolescence was more likely associated with epithelial ovarian cancer in adulthood. Nonetheless, no correlation between adult BMI and ovarian cancer risk was revealed (Engeland *et al*., 2003). Furthermore, a higher ovarian cancer risk has been demonstrated in premenopausal women who were overweight or obese compared to postmenopausal women (Liu *et al*., 2015; Reeves *et al*., 2007; Olsen *et al*., 2007).

The association between body size (height and BMI) and the risk of ovarian cancer was also investigated by the Collaborative Group on Epidemiological Studies of Ovarian Cancer in an individual participant meta-analysis including 47 Epidemiological Studies (Collaborative Group on Epidemiological Studies of Ovarian Cancer, 2012). Hormone therapy for menopause had a considerable effect on this association, indicating increased relative risk of ovarian cancer among obese women who never used hormone therapy, confirming the modifying effect of hormone therapy (Leitzmann *et al*., 2009). Besides, a dose-response meta-analysis of prospective observational studies assessed the association between adult weight gain and adiposity-related cancers, showing a 13% increase in the risk of developing ovarian cancer per every 5kg increase in weight gain in postmenopausal women with no- or low-hormone replacement therapy (Keum *et al*., 2015).

## OBESITY AND OVARIAN CANCER SURVIVAL

Ovarian cancer is a highly fatal disease due to its poor prognosis, with a 5-year survival rate of less than 50%, and a ten-year survival rate of about 35% (Sankaranarayanan & Ferlay, 2006; Baldwin *et al*., 2012). Its poor survival is mainly attributed to its insidious onset, resulting in high proportions of metastatic spread beyond the pelvis upon diagnosis (Protani *et al*., 2012; Howlader *et al*., 2011). Several known prognostic indicators of survival time have been indicated, like age at diagnosis, tumor grade, and success of debulking surgery (Protani *et al*., 2012; Yang *et al*., 2011).

Among potentially modifiable prognostic factors, obesity, and excess fat in adipose tissue have been found to result in the poor prognosis for gynecological cancers (Calle *et al*., 2003; Rodriguez *et al*., 2002; Pavelka *et al*., 2006; Zhang *et al*., 2005). It has been reported that ovarian cancer mortality can be affected by obesity influencing tumor biology (Bae *et al*., 2014).

Moreover, it's been progressively documented that adipose tissue is a crucial component of the ovarian cancer metastatic microenvironment, functioning as a lipid reservoir to maintain the high-energy demands of cancer cells (Nieman *et al*., 2011; Colvin, 2014). Tumor strongly affects ovarian cancer metastatic success through variations in lipid regulatory factors, increased vascularity, and decreased inﬁltration of M1 macrophages, leading to the negative correlation between obesity and ovarian cancer survival (Liu *et al*., 2015).

Triacylglycerols, which compose the main part of lipid species in adipose tissue of the normal human body, produce fatty acids during their synthesis or breakdown (Al-Sulaiti *et al*., 2018). Obesity promotes the hypertrophy of adipocytes within adipose tissue and accumulates the excess triacylglycerol (Al-Sulaiti *et al*., 2018). Triacylglycerols are also associated with the inflammatory cytokine IL-6 in adipose tissue (Al-Sulaiti *et al*., 2018). Notably, triacylglycerol is associated with higher risk of serous ovarian tumors (Zeleznik *et al.*, 2020). Dysregulation of lipid metabolism and the biosynthesis of triacylglycerol from fatty acids in ovarian cancer leads to the enhanced migration and metastasis of the cancer cells (Zeleznik *et al*., 2020). Moreover, evidence suggests that the level of circulating triacylglycerol may be a useful biomarker for ovarian cancer (Zeleznik *et al*., 2020).

In the study by Poole *et al*. (2016), it has been proposed that pre-diagnostic BMI, as one modifiable lifestyle factors, might affect the survival of ovarian cancer patients, and low pre-diagnostic BMI might be associated with a better prognosis. Moreover, a comprehensive meta-analysis ran by Bae *et al.* (2014) reported that despite the ambiguity of the connection between obesity at diagnosis and ovarian cancer patients' survival, obesity five years before the diagnosis of ovarian cancer and obesity at a young age were related to poor prognosis. They suggested that BMI at diagnosis cannot be used as a prognostic factor for ovarian cancer patients' survival (Bae *et al*., 2014). Likewise, a previous meta-analysis of studies reported higher mortality among those suffering from obesity during early adulthood or before diagnosis, although there was no correlation with obesity found around the time of diagnosis (Yang *et al*., 2011). Furthermore, an international collaborative analysis using the results from the Ovarian Cancer Association Consortium was undertaken to evaluate the association between pre-diagnosis BMI, progression-free survival, ovarian cancer-specific survival, and overall survival among women with invasive ovarian cancer. Intriguingly, the adverse relation between obesity and ovarian cancer survival seemed consistent regardless of the time of BMI measurement. Considering tumor histologic subtypes, positive but statistically non-significant associations were strongest for women with low-grade serous and endometrioid subtypes. In contrast, only the high-grade serous cancers revealed a borderline significant positive association with survival (Nagle *et al*., 2015). Furthermore, it has been reported that pre-diagnosis obesity could increase the risk of mortality in ovarian cancer patients (Zamorano *et al*., 2019). In general, compared with women within the normal-weight range, obese women showed poorer progression-free and overall survival. Therefore, it seems that retaining a normal BMI can be a powerful preventive tool.

Additionally, several studies have investigated the effects of BMI on surgical morbidity and clinical outcomes in ovarian cancer patients. A recent meta-analysis of five studies showed that obesity is linked with more wound complications and a longer hospital stay for these patients. However, there were no significant differences between obese and non-obese patients regarding other operative outcomes, including cytoreduction status, estimated blood loss, operation time, transfusion rates, and 30-day mortality (Smits *et al*., 2016). Besides, it was reported that weight loss therapy in the case of a 41-years old woman with end-stage ovarian cancer could improve cancer and transform it into small ovarian cysts (Oshakbayev *et al*., 2014). Therefore, elimination of obesity, as a modifiable factor, may prolong the life of ovarian cancer patients. However, it is noteworthy that a recent clinical study demonstrated that the pre-diagnostic physical activity of patients with ovarian cancer is not associated with mortality (Zamorano *et al*., 2019).

Chemoresistance is also a major problem in patients with ovarian cancer (Han *et al*., 2018). On the other hand, treatment strategies may also be different in obese patients with epithelial ovarian cancer. Obese patients are at a particular risk of receiving inappropriate low doses of chemotherapy, owing to toxicity concerns and dose capping practices, compromising their progression-free and overall survival. Chemotherapy dosing should be adjusted based on body surface area, using actual weight according to the existing clinical guidelines (Horowitz & Wright, 2015; Griggs *et al*., 2012).

However, although the studies mentioned above indicate that BMI can be associated with ovarian cancer survival, some studies have not found any evidence of this association (Tyler *et al*., 2012; Barrett *et al*., 2008; Fotopoulou *et al*., 2011). Thus, to elucidate the various effects of obesity on survival rates, we need more well-designed studies.

## MECHANISMS ASSOCIATING OBESITY TO OVARIAN CANCER

The related mechanisms contributing to increased risk of ovarian cancer incidence and mortality following metabolic impairments, including obesity, are not entirely understood. It has been proven that adipose tissue not only serves as calorie storage, but it is also a source of both pro-inflammatory and anti-inflammatory factors, known as adipocytokines. Dysregulation of adipokine and cytokine levels can be derived from excess adipose tissue, alter tissue immune responses, and help in tumor evasion of immune responses (Liu *et al*., 2015; Preston *et al.*, 2011). On the other hand, since adipose tissue acts as an endocrine organ that integrates various physiological processes, excess adiposity also causes altered endocrine function, leading to major alterations in pro-tumorigenic signal transduction pathways (Vrachnis *et al*., 2016). The most widely accepted biological mechanisms are discussed as follows:

### Adipocytokines

Cytokines are secreted proteins released by cells, and are involved in chemotaxis and cell growth. Elevated levels of adipocytokines, which are produced in adipose tissue [including IL-5 (Interleukin-5), IL-6, IL-8, IL-10, IL-12, IL-13, leptin, C reactive protein (CRP), IFNγ, monocyte chemotactic protein-1 (MCP-1), and TNF-α (tumor necrosis factor α)] (Schmidt *et al*., 2015; Wang *et al*., 2007) and numbers of immune cells (mainly macrophages), as well, have been shown in patients with obesity (Weisberg *et al*., 2003). It has been indicated that pro-inflammatory cytokine IL-6 is elevated in the serum of patients with ovarian cancer and it is related to poor outcomes (Lane *et al*., 2011). IL-6 activates the JAK-STAT3 pathway, and thereby promotes the invasion and metastasis of ovarian cancer cells (Kumar & Ward, 2014). Besides, IL-6 induces the Mcl-1 anti-apoptotic protein expression, which is recurrently overexpressed in ovarian cancer (Kolomeyevskaya *et al*., 2015), and it is associated with advanced tumor grade and poor survival in epithelial ovarian cancer (Chen *et al*., 2013). IL-6 is also accompanied by chemotherapy resistance, further proving the role of this cytokine in ovarian cancer outcomes (Gastl & Plante, 2001). IL-8 and its receptor (CXCR1) are upregulated in ovarian cancer cells and mediate homing, migration, and adhesion of ovarian cancer cells (Nieman *et al*., 2011). Besides, increased levels of TNF-α, contributes to insulin resistance, and has been associated with tumor grade and poorer survival in epithelial ovarian cancer (Kolomeyevskaya *et al*., 2015). Higher levels of CRP, as a marker of inflammation, have also been linked with the increased risk of developing ovarian cancer (Braun *et al*., 2011). There has been a close relationship between leptin, a hormone produced by immature adipocytes, and hormonal regulation of normal ovarian tissue. In addition, leptin is associated with estradiol secretion from the ovaries (Ray *et al*., 2018). Leptin usually regulates the energy balance of the body, but in obesity, leptin is involved in pro-inflammatory processes (Ray *et al*., 2018). Notably, the independent predictive value of leptin in combination with other analytes has been shown in ovarian malignancy (Mor *et al*., 2005). Leptin proliferative effects on ovarian cancer cell lines OVCAR-3 and A2780, after transfection with estrogen receptor-α, has also been described (Choi *et al*., 2011). While serum leptin levels are reduced in patients with ovarian cancer, the expression of leptin and its receptors are upregulated in ovarian cancer tissue (Ray *et al*., 2018). Overexpression of leptin receptors correlated with worse progression-free survival and contrastingly decreased levels of leptin have been reported with ovarian cancer progression (Kato *et al*., 2015; Uddin *et al*., 2009). Besides, inhibition of established tumorigenic effects of leptin via direct inhibition of the PI3K pathway (Choi *et al*., 2011; Uddin *et al*., 2009; Hoffmann *et al*., 2016), which is activated in ovarian cancer cells, has also been demonstrated by numerous studies (Keum *et al*., 2015; Chen *et al*., 2013; Hoffmann *et al*., 2016). Poorer outcomes in epithelial ovarian cancer were correlated with enhanced levels of leptin, as well as the expression of its receptor and the leptin to adiponectin ratio (Kato *et al*., 2015; Diaz *et al*., 2013). Leptin has been shown to contribute to metastatic advancement of epithelial ovarian cancer by assisting cell migration and tissue invasion by binding to OB-Rb mediated via JAK/STAT3, MAPK, AKT, mTOR, RhoA/ ROCK, and MYPT1 signaling pathways (Wu *et al*., 2012). Furthermore, leptin is involved in anti-apoptotic process through inhibition of some apoptosis pathway elements such as TNF receptor 1, caspase-6, caspase-3, and Bad (Ray *et al*., 2018). Leptin also enhances ovarian cancer progression through conservation of stem cells and mesenchymal characteristics of the cancer cells (Ray *et al*., 2018).

Adiponectin with insulin-sensitization, antiangiogenic, anti-inflammatory, and anti-neoplastic properties is reduced in obesity (Hada *et al*., 2007). Despite no correlation between adiponectin levels and progression of ovarian cancer (Jin *et al*., 2016), reduced serum adiponectin levels have been found in ovarian cancer (Jin *et al*., 2016; Otokozawa *et al*., 2015). Adiponectin anti-proliferative effects have mostly contributed to its induced reduction in the bioavailability of proinflammatory factors, performing crucial roles in the cancer-related metabolic syndrome (Booth *et al*., 2015). In a recent study in ovarian cancer, longer disease-speciﬁc survival (57 months) has been seen in women with low leptin to adiponectin ratios compared to those with medium or high levels (49 and 37 months, respectively).

Hyperactivation of the fatty acid oxidation pathway was reported in metastatic ovarian cancer cells (Zhu *et al*., 2019). Activated fatty acid oxidation signaling pathway contributes to the loss of NKX2-8 and reprogramming of fatty acid metabolism of ovarian cancer cells and results in chemoresistance (Zhu *et al*., 2019).

Relative hypoxia with low oxygen and nutrient availability are enhanced in the increased growth of individual adipocytes and adiposity (Park *et al*., 2014). Increased expression of leptin, IL-6, and VEGF (vascular endothelial growth factor) and decreased adiponectin have been reported to be caused by hypoxia (Preston *et al*., 2011). Indeed, this relative hypoxia upregulates the expression of hypoxia-inducible factor (HIF), a transcription factor, to mediate the survival adaptation of cancer cells (Park *et al*., 2014). HIF increases the expression of inflammatory cytokines (like IL-6, CXCR4), attracting macrophages that release inflammatory factors such as TNF-α and MCP-1 (Park *et al*., 2014). In epithelial ovarian cancer, upregulated CXCR4 has been shown to be accompanied by elevated recruitment of tumor-associated macrophages, associated with poor prognosis, and production of proangiogenic growth factors (Downs *et al*., 2002).

The salt-inducible kinases 2 (SIK2), a member of the AMP-activated protein kinase (AMPK) family, is produced specifically in the adipose tissue and plays roles in the modulation of different biological processes such as adipocyte energy metabolism and macrophage signaling pathways (Du *et al*., 2008). SIK2 enhances inflammation through the downregulation of anti-inflammatory cytokines such as IL-10 and upregulation of inflammatory cytokines such as IL-6, IL-12, TNF-α (Kargbo, 2018). It has been reported that SIK2 expression is elevated in obesity and insulin resistance (Säll *et al*., 2017). Moreover, SIK2 involves various disorders, especially ovarian cancer, by different proliferative and anti-apoptotic mechanisms (Gao *et al*., 2020). Hence, it has been proposed that SIK2 is a crucial oncogenic element in human ovarian cancer (Gao *et al*., 2020). SIK2 enhances the level of HIF-1α through activation of the PI3K/AKT signaling pathway and also activates Drp1 phosphorylation-mediated mitochondrial fission and promotes metabolic reprogramming to switch from oxidative phosphorylation to glycolysis, which is known as Warburg effect, in ovarian cancer cells to supply cellular energy for the progression and metastasis of the cancer cells (Gao *et al*., 2020). Recently, AMPK has been identified as a crucial energy regulator in ovarian cancer cells (Chen *et al*., 2019). In other words, ovarian cancer cells provide energy from lipid metabolism through AMPK/ACC/FASN lipogenesis and AMPK/TAK1/NF-κB signaling (Chen *et al*., 2019).

Secreted protein acidic and rich in cysteine (SPARC or BM-40) is an extracellular matrix protein and has the inhibitory effects on the differentiation of the adipocytes and adipogenesis (Nie & Sage, 2009). Although the precise roles of SPARC in tumors are not still obvious, dysregulation of this protein has been reported in obesity and diabetes (Kos & Wilding, 2010; Kos *et al*., 2009). It has been shown that SPARC-null mice were more likely to be affected by Diet-induced obesity (Nie *et al*., 2011). This protein has anti-inflammatory properties and tumor suppressor effects in ovarian cancer through inhibition of the metabolic plasticity and mitochondrial bioenergetics; thereby, SPARC inhibits the cancer cell interactions, proliferation, and invasion of ovarian cancer (John *et al*., 2019; Naczki *et al*., 2018). It has been suggested that SPARC exerts the tumor suppressor effects in ovarian cancer through inhibition of cEBPβ, NFkB, AP-1, and their downstream inflammatory effects (John *et al*., 2019).

### Hormones

Hormones such as insulin, IGF-1 (Insulin-like growth factor 1), and IGF-2 are able to activate HIF-1 in conditions of decreased oxygen availability (Park *et al*., 2014). Cell growth in ovarian cancer cells has been shown to be promoted by IGF-1, which is usually related to obesity and hyperinsulinism (Hursting *et al*., 2003). The associated increase in IGF-1 and its signaling in obesity and DM is inversely linked with the survival of epithelial ovarian cancer (Spentzos *et al*., 2007). Furthermore, metabolic derangements induce significant changes in sex hormones, which probably play a mechanistic role in ovarian cancer progress (Park *et al*., 2014). Circulating aromatase-derived estrogen is positively connected to BMI in postmenopausal women (Cowey & Hardy, 2006), so that every 5 unit increase in BMI leads to a 12.8% increase in unconjugated estradiol levels (Schairer *et al*., 2016). Also, circulating levels of estrogen and androgen have been revealed to be increased by obesity mostly in postmenopausal women (McTiernan *et al*., 2006). Estradiol, probably by promoting the proliferation of ovarian epithelial cells (Cunat *et al*., 2004), was associated with the endometrioid subtype of ovarian cancer (Schock *et al*., 2014), which is more likely estrogen and progesterone receptor-positive (Hecht *et al.*, 2009). Additionally, despite the more obvious role of estrogen exposure in uterine carcinogenesis, the contribution of increased estrogen and estrogen signaling in ovarian cancer development has also been documented (Laws *et al*., 2014). There are also some studies supporting the evidence that ovarian cancer risk may be increased by higher circulating androgen levels (Lukanova *et al*., 2003; Schock *et al*., 2014). As such, the higher risk of ovarian cancer in polycystic ovarian syndrome has been documented (Barry *et al*., 2014; El Hayek *et al*., 2016). It should be noted that SIK2, which is increased in obesity and related to ovarian cancer, also plays roles in hormonal signal transduction in adipose tissue (Gao *et al*., 2020; Katoh *et al*., 2004).

## DIABETES MELLITUS AND OVARIAN CANCER

DM is a frequently detected metabolic disease. The disease features a lack of insulin secretion ability (type 1) and inefficiency in insulin use (type 2) (CDC, 2017). At present, there are over 425 million people diagnosed with DM living in the world. In 2017, more than 727 billion US dollars were spent in facilities associated with DM treatment and healthcare, and such spending has been expected to rise progressively, and it may exceed 800 billion within 25 years in the future (CDC, 2017).

The inflammatory situation and increased number of immune cells in obesity can impair adipose tissue function, causing insulin resistance and type 2 DM, and eventually create a tumorigenic environment (Ray *et al*., 2018). Several epidemiologic studies and meta-analyses confirmed the theory of the direct effect of DM in elevated risk of colorectal, breast, and endometrial cancers (Vigneri *et al*., 2009), and they have shown that it is related with poor survival in colon, pancreas, and breast cancers (Coughlin *et al*., 2004). These consequences appear to be unrelated to obesity (Coughlin *et al*., 2004), as a recognized risk factor for both the progress of cancer and mortality from it (Calle *et al*., 2003; Barone *et al*., 2008; Craig *et al*., 2016). High serum levels of glucose in DM may be associated with poor prognosis for ovarian cancer and less survival of the patients (Lamkin *et al*., 2009). Furthermore, triacylglycerol is associated with DM and also the higher risk of serous ovarian tumors (Zeleznik *et al*., 2020). Hence, disrupted triacylglycerol levels in DM patients may enhance the metastasis of ovarian cancer cells.

## MECHANISMS ASSOCIATING DIABETES MELLITUS TO OVARIAN CANCER

The association of DM and cancer is a multipart process. The molecular perspective suggests that elevated IGF-1, increased cytokine and estrogen levels, adipokine imbalances, and hyperinsulinemia contribute to a high risk of malignancy besides leading to poorer patient outcomes (Howe *et al*., 2013).

The etiological relation of DM and ovarian cancer is not obvious, but there is sufficient biological evidence. Overexpression of IGF-I and -II are demonstrated in various cancers (Gallagher & LeRoith, 2015; Malaguarnera & Belfiore, 2014), which may result in elevated proliferation, besides invasion and metastasis pathways (Gallagher & LeRoith, 2011; Pollak, 2012). Increased levels of IGF-I and -II have been linked with a reduction in ovarian cancer survival (Sayer *et al*., 2005; Yu & Rohan, 2000). Furthermore, insulin resistance and DM are associated with low serum sex hormone-binding globulin (Wallace *et al*., 2013; Ye *et al*., 2017), which can cause increased free estrogen production. The carcinogenic evidence of high estrogen levels is indubitable in endometrial and ovarian cancer (Laws *et al*., 2014; Brown & Hankinson, 2015). An addition, convincing association of DM and cancer progress is through inflammatory pathways. Adipose metabolic dysregulation as a hallmark of DM (Amato *et al*., 2014), can result in high levels of inflammatory cytokines, including IL-6 and TNF-α (Amato *et al*., 2014). These cytokines stimulate cell proliferation, invasion, and evasion of antitumor immunity pathways (Yu *et al*., 2009).

SIK2 is also elevated in DM and plays roles in modulating the insulin-signaling cascade by phosphorylation of Ser-794 of insulin-receptor-substrate-1 (IRS-1) (Säll *et al*., 2017). TBC1D8, an activated factor of the GTP enzyme, involved in DM (Frasa *et al*., 2012), participates in ovarian cancer tumorigenesis and metabolic reprogramming (Chen *et al*., 2019). TBC1D8 is unregulated in the aggressive ovarian cancer cells and it is associated with poor prognosis in these patients (Chen *et al*., 2019). SPARC, which has anti-inflammatory and inhibitory effects in ovarian cancer cells, is also related to obesity-induced insulin resistance (Harries *et al*., 2013). Dysregulation of SPARC alters the levels of inflammatory cytokines related to insulin-stimulated glucose transport, glucose transporter 4, and ATP synthesis in mitochondria, and eventually induces insulin resistance (Shen *et al*., 2014).

It has been stated that the increased level of insulin, which is common in type 2 DM due to insulin resistance, causes leptin overproduction, which in turn, increases leptin concentration, is related to the risk of incidence and progression of endometrial and ovarian cancers (Doucet *et al*., 2000; Ma *et al*., 2013; Jin *et al*., 2016). Moreover, leptin can also lead to vascular VEGF overexpression, a feature of malignant tumor development (Gonzalez-Perez *et al*., 2010). In addition, hyperinsulinemia enhances the levels of estradiol and testosterone, and thereby leads to poor prognosis in cancer patients (Ruge *et al*., 2012). On the other hand, insulin may have an anti-apoptotic effect on the cancer cells by alteration in the PI3K/AKT pathway and mitotic kinase pathway (Gryko *et al*., 2014).

Recently, microRNAs (miRNAs) have been proposed as the molecules, which can be potentially involved in ovarian cancer in obese and diabetic women. MiR-150 is suggested as a miRNA which its deficiency is involved in the obesity-associated inflammation of adipose tissue and the resulting insulin resistance (Ying *et al*., 2016). Since miR-150 can upregulate glycolysis via targeting AKT3, it may directly reduce the Warburg effect and resistance in ovarian cancer cells (Yu *et al*., 2019; Wuerkenbieke *et al*., 2015). Moreover, miR-29b, which is dysregulated in obesity and DM, can also affect the Warburg effect via downregulating AKT2 and AKT3 in ovarian cancer cells (Dooley *et al*.,2016; Teng *et al*., 2015). So, miR-29b can negatively affect tumor glucose metabolism and thereby reduce the progression of ovarian cancer (Teng *et al*., 2015).

Hyperglycemia, as the most noticeable clinical mark of DM, involves tumor development via various pathways leading to increased proliferative, anti-apoptotic, and metastatic cancer activity (Masur *et al*., 2011; Li *et al*., 2011). In addition, hyperglycemia may cause endothelial dysfunction, endothelial cell death and aberrant neoangiogenesis (Bhattacharyya *et al*., 2012; De Mattia *et al*., 2008). Hyperglycemia-associated AGEs (advanced glycation end-products) promote pathological activation of protein kinase C, which can cause altered vascular proliferation. The interaction of AGEs with their receptors causes oxidative stress and inflammation, leading to cancer progression (Giorgi *et al*., 2017; Rojas *et al*., 2010).

Investigations regarding the effects of metformin proposed an improvement in both cancer risks, along with better survival (Habib & Rojna, 2013; Martin & Marais, 2012; Rattan *et al*., 2012). The metformin mechanism of action is not clear, even though inhibition of the mTOR pathway may participate in the metformin's anti-proliferative effects (Cantrell *et al*., 2010). There are observational and preclinical studies signifying a valuable influence of metformin on ovarian cancer survival (Dilokthornsakul *et al*., 2013; Garrido *et al*., 2018; Kheirandish *et al*., 2018).

## CONCLUSION

**Figure 1:**
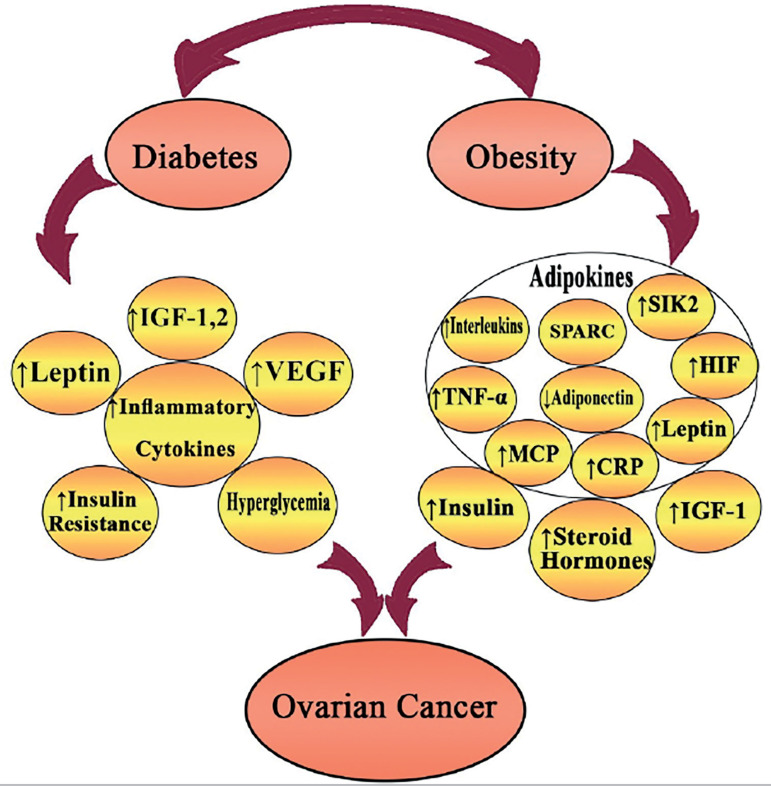
Figure showing the mechanism by which obesity and diabetes affect ovarian cancer.

The association between increased risk for developing ovarian cancer and mortality from it with obesity and DM has been highlighted in numerous studies (Figure 1). Considering the high and growing prevalence of obesity and DM, a comprehensive intervention on these metabolic abnormalities may diminish the worldwide burden from ovarian cancer. The prognostic significance of metabolic abnormalities may have considerable implications because of the feasibility of available interventions. Moreover, due to the lack of enough studies estimating the subtype-specific associations with metabolic abnormalities, supplementary studies are needed to overcome this problem.
